# Annual Commencment of the “Pennsylvania College of Dental Surgery”

**Published:** 1863-04

**Authors:** 


### Correspondence.

MESSRS. EDITORS:

The annual Commencement of the "Pennsylvania College of Dental Surgery" was held at the Musical Fund Hall, in this city, on the evening of February 27, 1863, in the presence of a large and fashionable audience.

There were *twenty* graduates out of a class of *forty-one*, representing thirteen States of the Union, and Cuba, Germany and Turkey.

The Valedictory Address was by Professor Buckingham, and abounded in appropriate and well timed remarks.

The Demonstrator's report gives the various operations performed upon seven hundred and seventy-two patients, among which are gold fillings, five hundred and fifty; and tin fillings, five hundred and twenty-six.

In the mechanical department, ninety-five patients were treated, and twelve hundred and forty-two teeth mounted.

The class was a very creditable one, and considering the times, was quite as large as could have been expected.

An attempt is now being made to obtain a charter from the Pennsylvania Legislature for another dental college, to be located in Philadelphia. The parties *immediately* interested in this movement, (or at least one or two of them who are at the bottom of it, and who no doubt expect to be leading members of the faculty,) are not influenced by a proper spirit, nor is the movement made from a pure desire to benefit the cause of dental education, though such be claimed for it, but rather from a spirit of antagonism, and to gratify a personal pique—to rule or ruin—affording a striking and exact illustration, in a petty way, of the spirit and principle of *secession.*

None intimately acquainted with the interests involved, and without prejudice, could bring himself to believe that the cause of dental education can be benefitted, or Philadelphia profited by the antagonism of another school; one must give way, for there is not, and can not be for the present, support for two schools in this city.

The efforts put forth to accomplish their purposes are worthy a better cause; a member of the Legislature, a practicing dentist, and *a corporator in the bill*, is applied to, and against his convictions, is argued into compliance, and consents to make a personal matter of it, and ask its passage of his fellow members on personal grounds, and thus it is "put through" the House. Another follows the bill into the committee of the Senate, urging its passage there. Petitions are prepared, and signatures procured of people who know nothing about denistry, or of the existence of the present school or no appreciation of either; and thus Legislators are made to believe that a great educational want exists, and to look with favor upon a measure for which there is really no public or professional necessity.

I am glad to have learned, incidentally, that the school in your city is in a more promising condition than ever before. When our national troubles are settled, there is nothing to prevent it from taking a position of greatly extended influence and usefulness.

Yours, O. U. C.

PHILADELPHIA, March 25, 1863.

